# Long-term follow up of human T-cell responses to conserved HIV-1 regions elicited by DNA/simian adenovirus/MVA vaccine regimens

**DOI:** 10.1371/journal.pone.0181382

**Published:** 2017-07-18

**Authors:** Nathifa Moyo, Nicola J. Borthwick, Edmund G. Wee, Silvia Capucci, Alison Crook, Lucy Dorrell, Tomáš Hanke

**Affiliations:** 1 The Jenner Institute, Nuffield Department of Medicine, University of Oxford, Oxford, United Kingdom; 2 Department of Pharmacy and Biotechnology, University of Bologna, Bologna, Italy; 3 NDM Research Building, Nuffield Department of Medicine, University of Oxford, Oxford, United Kingdom; 4 Oxford NIHR Biomedical Research Centre, University of Oxford, Oxford, United Kingdom; 5 International Research Center for Medical Sciences, Kumamoto University, Kumamoto, Japan; George Washington University, UNITED STATES

## Abstract

**Background:**

Durability of vaccine-elicited immune responses is one of the key determinants for vaccine success. Our aim is to develop a vaccination strategy against the human immunodeficiency virus type 1 (HIV-1), which induces protective and durable CD8^+^ T-cell responses. The central theorem of our approach is to focus T cells on highly conserved regions of the HIV-1 proteome and this is achieved through the use of the first-generation conserved vaccine immunogen HIVconsv. This immunogen vectored by plasmid DNA, simian adenovirus and poxvirus MVA was tested in healthy, HIV-1-negative adults in UK and induced high magnitudes of HIVconsv-specific plurifunctional CD8^+^ T cells capable of *in vitro* HIV-1 inhibition. Here, we assessed the durability of these responses.

**Methods:**

Vaccine recipients in trial HIV-CORE 002 were invited to provide a blood sample at 1 and 2 years after vaccination. Their PBMCs were tested in IFN-γ ELISPOT, 25-analyte Luminex, CFSE proliferation and intracellular cytokine staining assays, the last enhanced by HLA-peptide dextramer analysis.

**Results:**

12/12 (1 year) and 8/8 (2 years) returning subjects had median (range) of 990 (150–2495) and 763 (70–1745) IFN-γ SFU/10^6^ PBMC specific for HIVconsv, respectively, and recognized 5 (1–6) out of 6 peptide pools at 2 years. Over one-half of the HIVconsv–specific cells expressed at least 3 functions IFN-γ, TNF-α and CD107a, and were capable of proliferation. Among dextramer-reactive cells, naïve, transitional, effector and terminally differentiated memory subsets were similarly represented.

**Conclusions:**

First generation HIVconsv vaccine induced human T cells, which were plurifunctional and persisted for at least 2 years.

**Trial registration:**

ClinicalTrials.gov NCT01151319

## Introduction

A truly efficacious vaccination should elicit life-long immunity in vaccine recipients [1]. Such long-lasting protection may require concerted actions of both antibodies and CD8^+^ cytotoxic T lymphocytes (CTL), and will depend on the induction and maintenance of protective levels of immune memory, which can upon exposure to incoming infection either directly or following a rapid expansion exert effector functions [2]. Requirements for immunity against infections and/or subsequent disease are rarely well defined. While defence against different pathogens in general utilizes common mechanisms, in detail protective effector functions differ from pathogen to pathogen [3–7].

Our aim is to develop a vaccination regimen, which induces effective CD8^+^ T-cell responses against human immunodeficiency virus type 1 (HIV-1) [8, 9]. In humans, indirect evidence for the protective role of CD8^+^ T cells against HIV-1 comes from the temporal association of their expansion and resolution of primary viremia [10–15], extensive virus escape in targeted epitopes [12, 16–18] association of certain HLA class I allotypes with good clinical outcomes [11, 16, 17, 19–21] and identification of protective CD8^+^ T-cell epitopes in antiretroviral treatment (ART)-naïve patients [22–24]. Model infection of rhesus macaques with simian immunodeficiency virus (SIV) provided a direct demonstration that CD8^+^ cell depletion in infected macaques resulted in increased viremia [25, 26]. More recently, vaccines vectored by engineered molecular clone 68.1 of rhesus cytomegalovirus controlled [27–30] and eventually cleared [31] SIV infection in over half of experimentally challenged animals in the absence of SIV-specific antibody responses. Thus, vaccine induction of highly effective CTL could significantly contribute to reducing the acquisition of HIV-1 by complementing broadly neutralizing antibodies and may be central to HIV cure by limiting or even eliminating rebound viremia.

No simple functional or phenotypic T-cell marker has been consistently associated with HIV-1 control. This is because antigen-specific CD8^+^ T cells are a heterogeneous population capable of performing multiple functions and, in natural HIV-1 infection, CTL target both protective and non-protective epitopes [22–24], which further blurs any simplistic association attempts. To be beneficial, CD8^+^ T cells will have to display individually and as a population multiple attributes including specificity, breadth, quality, quantity, location and timing [32, 33]. We argue that all these features have to be right at the same time and if any one of them is suboptimal, the T cells/vaccine will fail to protect [8, 24]. Key parameters include specificity for protective epitopes [22–24], parallel recognition of multiple protective epitopes [9, 34, 35], optimal interaction with HLA-presented peptides [36], rapid expansion upon exposure to cognate antigens to reach protective frequencies [37, 38], killing of infected cells and production of soluble antiviral and intercellular signalling molecules [37–40]. Of these, IFN-γ promotes an antiviral state by converting the constitutive proteasome to the immunoproteasome [41], and upregulates the transporter associated with antigen processing (TAP) proteins [42, 43] and HLA class I [44, 45]. While measuring frequencies of IFN-γ-producing cells serves as an indicator for the presence of a response and a useful comparator of vaccine performances, it cannot be used alone for inferring anti-HIV-1 capacity of T cells. Therefore, other functions are frequently measured in the context of HIV-1 and vaccination such as TNF-α, which promotes apoptosis, inflammation and immunity [46–48], IL-2, which is the primary growth factor of T cells [49], and cytotoxicity, which is likely the most important function of CD8^+^ T cells, that can be assessed indirectly by granzyme and perforin cell content [50, 51] and surface expression of lysosomal-associated membrane protein 1 (LAMP-1)/CD107a as a marker of degranulation [52, 53]. Interestingly, the Fas-Fas ligand interaction, the alternative trigger of target cell killing to perforin/granzymes [54], is thought not to be important for killing of HIV-1-infected cells [51]. Clearly, measuring multiple functions simultaneously provides a more sensitive and complete evaluation of both natural infection- and vaccine-induced T-cell responses.

The central hypothesis of our T-cell vaccine strategy against HIV-1 is that focusing vaccine-elicited T cells on the conserved regions (not full-size proteins and not epitopes as a string-of-beads) of HIV-1 proteins will efficiently target both founder/transmitted and reactivated viruses, and if escape mutations in these regions occur, these will restrict the mutant virus replicative fitness [8, 24, 55]. CTL responses to such conserved epitopes are typically subdominant in natural infection, whereby immunodominance hierarchy often undermines their protective potential by precluding their efficient induction [56–60].

The first generation HIV-1 conserved immunogen HIVconsv was constructed [55] and presented to the immune system using DNA, engineered non-replicating simian adenovirus and non-replicating poxvirus modified vaccinia virus Ankara (MVA). So far, these vaccines have been tested in 8 phase I/IIa clinical trials in both HIV-1-negative and positive subjects. High frequencies of conserved region-specific CD8^+^ T cells were induced, which inhibited *in vitro* replication of four major HIV-1 clades ([61–67] and unpublished). Furthermore, 5 out of 13 HIV-1-positive subjects in trial BCN 02 controlled virus rebound following monitored antiretroviral treatment pause beyond the typical four-week period [66]. A number of novel CD8^+^ T-cell determinants were defined in HIVconsv vaccine recipients, which will inform future T-cell vaccine improvements and increase the power of early prediction of vaccine success or failure [63]. Here, we characterize the HIVconsv vaccine-induced T-cell responses in HIV-1-negative adults enrolled into trial HIV-CORE 002 [68] up to 2 years after the vaccine administration and show persistence of plurifunctional HIV-1-specific T cells of structured memory subsets.

## Materials and methods

### HIV-CORE 002 trial

Phase I/IIa trial HIV-CORE 002 was approved by the National Research Ethics Service (NRES) Committee West London (Ref: 10/H0707/52) and the UK Medicines and Healthcare products Regulatory Agency (Ref: 21584/0271/001). The extended follow up of the original trial HIV-CORE 002 was carried out under an approved Amendment to the Clinical Protocol. The study was conducted according to the principles of the Declaration of Helsinki (2008) and complied with the International Conference on Harmonization Good Clinical Practice guidelines. All volunteers gave written informed consent before participation. Tissue samples were stored in the Oxford Vaccine Centre Biobank in compliance with the UK Human Tissue Act 2004 and with approval from local NRES (Ref: 10/H0504/25).

### Isolation and cryopreservation of PBMCS

Blood was drawn into heparinized vacutainers (Becton Dickinson) and processed by the laboratory within 6 hours. Standard procedures were used for cryopreservation [68].

### Peptides and antigens

HIVconsv peptides (Ana-Spec, San Jose, USA) and their truncated versions (GenScriptHK, Hong Kong) were reconstituted to 10–40 mg/ml in DMSO and diluted to working stock solutions of 4 mg/ml in PBS. Recognized Gag- and Pol-derived peptides were assembled into personalized pools as described previously [68].

### *Ex vivo* IFN-γ ELISPOT assay

Freshly isolated PBMCs were used in an IFN-γ ELISPOT assay as described previously [68]. ELISPOT plates (S5EJ044I10; Merck Millipore) pre-wetted for 1 min with 15 μl of 35% ethanol were coated overnight at 4°C with anti-IFN-γ antibody (10 μg/ml in PBS; clone 1-D1K; Mabtech). Prior to use, plates were washed with PBS and blocked with R10 (RPMI 1460 supplemented with 10% FBS, 2 mM L-glutamine, 1 mM sodium pyruvate, 10 mM HEPES and penicillin-streptomycin antibiotics; Sigma Aldrich) for a minimum of 1 hour at 37°C. The PBMCS were plated out at 2x10^5^ cells/well in 50 μl. For HIVconsv, Pools 1–6 responses were detected in triplicate wells. Six negative no-peptide control wells were cells cultured in R10 supplemented with 0.45% DMSO. Positive controls in triplicate wells were cells cultured with 10 μg/ml PHA (Sigma Aldrich) or a pool of FEC peptides at 1 μg/ml. As an external positive control, the cell line NKL was cultured in duplicate wells in the presence of 4 μg/ml PMA plus 1 μg/ml ionomycin (both from Sigma Aldrich), The cells were incubated overnight at 37°C in 5% CO_2_. Spots were visualised using biotin-conjugated anti-IFN-γ mAb combined with alkaline phosphate-conjugated streptavidin (both from Mabtech) and the colour was developed using substrate BCIP/NBT^Plus^ (Mabtech). The reaction was stopped after 5 min by washing under the tap. The plates were air dried overnight and the spots counted using an AID ELISpot Reader and version 5.0 software (AID GmbH). The median number of spot-forming units (SFU) in no-peptide wells were subtracted from test wells and the results were expressed as the median net SFU/10^6^ PBMC.

### CSFE proliferation assay

Cryopreserved PBMC were thawed, resuspended in pre-warmed PBS with 0.1% BSA at a final concentration of 10^6^ cells/ml and labelled with 750 nM CFSE (5(6)-Carboxyfluorescein diacetate *N*-succinimidyl ester; Molecular Probes^™^) for 10 min at 37°C, 5% CO_2_. The staining was quenched by adding 5 volumes of ice-cold R10 followed by a 5-min incubation on ice. The cells were pelleted, washed and plated in 96-well round-bottom plates at a concentration of 1 x 10^6^ cells/well. The CFSE-labelled cells were then stimulated with 1.5 μg/ml of each peptide in personalized pools or 1 μg/ml SEB (positive control) and R10 (negative control) for 5 days, stained with a dead cell marker (LIVE/DEAD Fixable Aqua stain; Invitrogen) and anti-CD4-BV605 (BioLegend), anti-CD3-ECD (Beckman Coulter) and anti-CD8-Aleva Fluor 700 (eBioscience) mAbs, fixed and acquired on a BD LSR II flow cytometer. Data analysis was performed using FlowJo software (Tree Star Inc.) with gating shown in S1 Fig.

### Luminex assay

Luminex is a multiplex bead array that measures multiple cytokine and chemokine production in a single sample of culture supernatant. PBMCS were thawed and adjusted to 5 x 10^6^ cells/ml in tissue culture medium containing either mapped-positive peptide pools at 1.5 μg/ml per peptide, staphylococcus enterotoxin B (SEB; Sigma-Aldrich) at 1.0 μg/ml or R10 media as a negative control together with anti-CD28 and anti-CD49d mAbs both at 1 μg/ml in a final volume of 200 μl per well. The plates were incubated for 48 hours at 37°C, 5% CO_2_, and 150 μl of supernatant was removed from each well and stored at -80°C until use [69]. A human pre-mixed multi-analyte kit (Magnetic Luminex screening assay, R & D Systems Ltd) was used to measure the following analytes; IFN-γ, TNF-α, TNF-β, IL-2, IL-3, IL-5. IL-6, IL-13, IL-17A, IL-17E, IL-17F, IL-27, SDF-1α (CXCL12), IP-10 (CXCL10), MIP-1α (CCL3), MIP-1β (CCL4), MIP-3α (CCL20), RANTES (CCL5), FasL (CD95L), GMCSF, Granzyme A, Granzyme B, MIG (CXCL9), Fas (CD95) and CD40L. The culture supernatants were diluted 1:1 and assayed in duplicate according to the kit instructions. The plate was read using Luminex 200 and XPONENT softwares. Levels seen in unstimulated wells were used as background controls.

### Intracellular cytokine staining (ICS) assay

PBMCS were thawed and stimulated with either mapped-positive peptide pools [68] at 1.5 μg/ml per peptide, SEB at 1.0 μg/ml or tissue culture media as a negative control. The cultures were supplemented with anti-CD28 and anti-CD49d mAbs (Becton-Dickinson) both at 1.0 μg/ml and with anti-CD107a PE-Cy7-conjugated mAb (Becton-Dickinson). The cells were incubated at 37°C, 5% CO_2_ for 2 hours prior to the addition of Brefeldin A and monensin (Becton-Dickinson) and then left in culture overnight. For HLA-dextramer staining the cells were centrifuged briefly and the pellet re-suspended in 100 μl of PBS plus 5% BSA (Sigma-Aldrich) plus 5 μl of HLA-A*02:01 dextramer conjugated to PE (Immudex, Copenhagen). The cells were incubated at room temperature in the dark for 10 min. A mastermix of anti-membrane marker mAbs was prepared containing LIVE/DEAD fixable aqua stain (Molecular Probes, Invitrogen), CD8 eFluor 780 (eBiosciences), CD16 BV650, CD14 BV650, CD19 BV650 and CD4 PE-Cy5 (all from Biolegend) and 100 μl added to each tube. The cells were incubated at 4°C for 30 min and then permeabilized using Fix/Perm solution (Becton-Dickinson) for 20 min at 4°C. The cells were washed with Perm Wash buffer (Becton Dickinson) and a mastermix of anti-intracellular molecule mAbs was prepared containing CD3 ECD (Beckman Coulter), TNF-α FITC (eBiosciences), IFN- γ V450 (Becton-Dickinson), IL-2 BV605 and Perforin APC, clone B-D48, which recognizes a determinant expressed on newly synthesized perforin [70] (Biolegend). The cells were incubated at 4°C for 30 min, washed and fixed with 1% paraformaldehyde in PBS prior to running on an LSRII flow cytometer (Becton-Dickinson).

### Memory assay

PBMCs were thawed, stained with 5 μl of the HLA-A*02:01 dextramer conjugated to PE for 10 minutes at room temperature, followed by the addition of 100 μl of a mastermix of anti-membrane marker mAbs containing LIVE/DEAD fixable aqua stain (Molecular Probes, Invitrogen), CD3 ECD (Beckman Coulter), CD4 BV605 and CCR7 Pacific blue (Biolegend), CD8 Alexa Fluor 700, CD14 PE-Cy7, CD16 PE-Cy7, CD19 PE-Cy7, CD45RA APC, CD57 FITC, TIGIT PerCP-eFluor710, PD-1 APC-eFluor780 (eBiosciences) and CD27 Qdot 655 (Life Technologies). The cells were incubated at 4°C for a further 20 minutes, washed and fixed with 1% paraformaldehyde in PBS prior to running on an LSRII flow cytometer (Becton-Dickinson).

### IFN-γ capture assay

PBMCs were thawed, stimulated for 3 hours at 37°C with either mapped-positive peptide pools at 1.5 μg/ml per peptide, SEB (Sigma-Aldrich) at 1 μg/ml or tissue culture media as a negative control. The cells were washed with PBS pH 7.2 plus 0.5% BSA and 2 mM EDTA, labelled with 10 μl of IFN-γ catch reagent for 5 minutes at 4°C followed by the addition of 1 ml of warm media and incubated at 37°C for 45 minutes on a tube rotator (VWR). Immediately following incubation, cells were placed on ice for 5 minutes, washed and stained at 4°C for 10 minutes with 100 μl of a mastermix containing IFN-γ PE (Miltenyi Biotec) and the anti-membrane marker mAbs described above in the memory assay protocol. Cells were washed and fixed with 1% paraformaldehyde in PBS prior to running on an LSRII flow cytometer (Becton-Dickinson). All antibodies were used at pre-titrated, optimal concentrations. For analysis, the following gating strategy was used (S1 and S2 Figs): FSC v SSC lymphocyte gate, FSC v LIVE/DEAD viability gate, FSC v BV650 negative dump gate, FSC-W v FSC-A doublet gate, CD3 T-cell gate, CD4 vs CD8 single CD4 or CD8 gate followed by each of TNF-α, IFN-⅟, CD107a and IL-2 on the T-cell subsets. Boolean gating analyses were used to determine plurifunctional T-cell subsets (FlowJo). To examine HLA-peptide dextramer reactivity, a CD8 vs PE analysis gate was added and Boolean gates set up for the intracellular cytokines. The anti-perforin mAb clone used in this study cannot distinguish newly synthesized from pre-formed perforin [70] and so perforin expression was measured in cells that also expressed either TNF-α, INF-γ, CD107a or IL-2 again using Boolean gating (S2 and S3 Figs).

### Statistics

Analyses of variance were performed using GraphPad Prism 6.0d. ELISPOT and flow cytometry results were assumed to be non-Gaussian in distribution, thus non-parametric tests were used throughout and medians (range) are shown. Variation among groups was assessed in the Kruskal-Wallis test and individual group means were compared either to control using Dunn’s multiple comparison or among themselves in pairwise comparisons followed by the Bonferroni adjustment. For unpaired analyses, the Mann-Whitney U test was used. Correlations were made using Spearman rank test. Two-tailed *p* values were used and *p* value of less than 0.05 was considered statistically significant.

## Results

### Vaccine-elicited HIVconsv-specific responses were readily detected after 2 years

**Fig 1:**
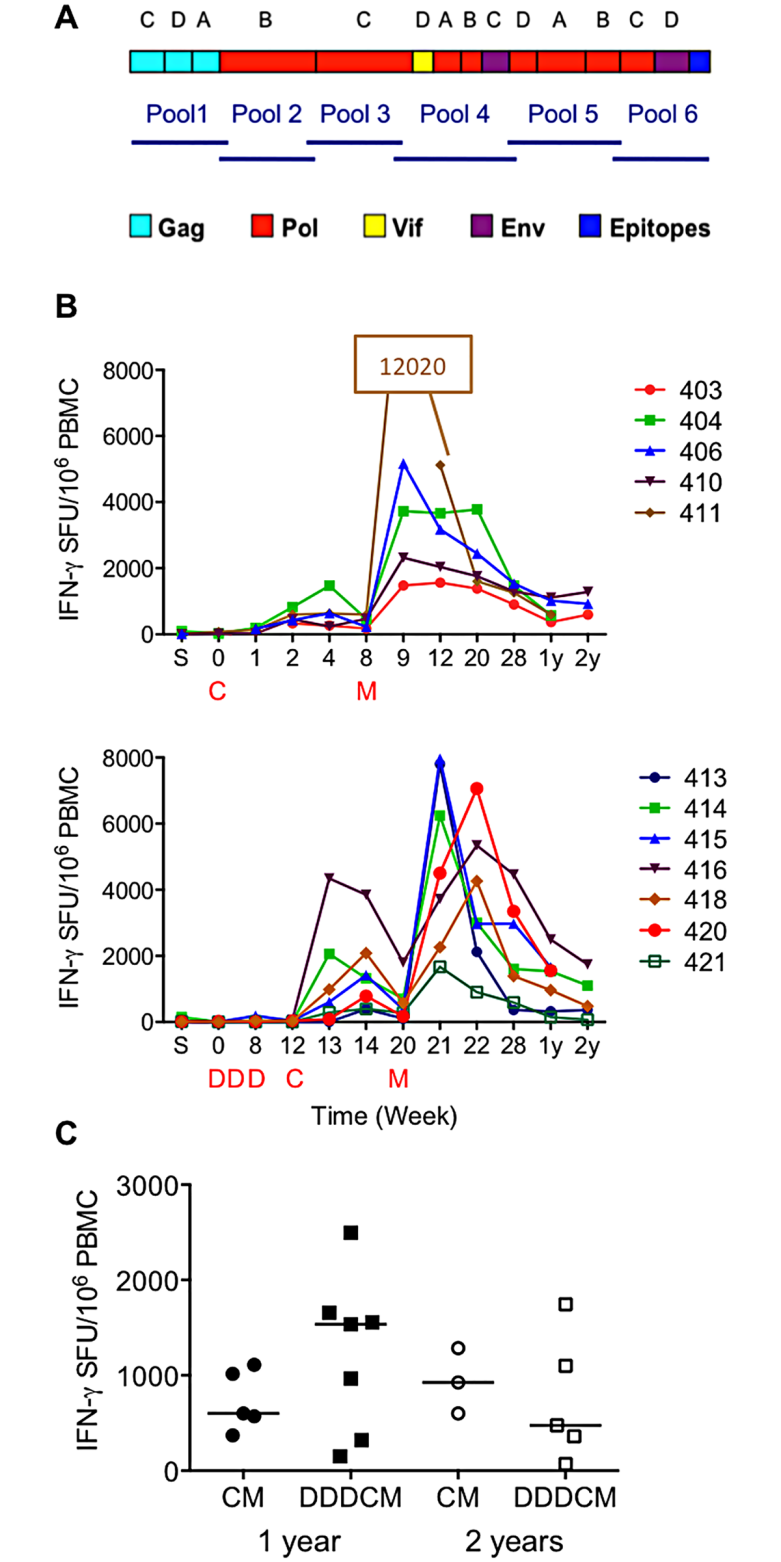
HIVconsv vaccine-induced human T-cell responses. (A) Schematic representations of the HIVconsv immunogen and six pools of a total of 199 overlapping peptides used for the IFN-γ ELISPOT assay. HIVconsv is a chimeric protein assembled from 14 highly conserved regions of the HIV-1 proteome, the HIV-1 protein origins of which are colour-coded below. Each of the regions uses a consensus amino acid sequence of the HIV-1 clade indicated above the schematics. The C-terminal epitopes include Mamu-A*01- and H-2^d^-restricted immunodominant CTL epitopes and a Pk tag recognized by a monoclonal antibody, which together facilitate the quality control of the vaccines. (B) Fresh *ex vivo* net total (sum of six pools) IFN-γ ELISPOT assay frequencies of HIVconsv-specific T cells of returning healthy HIV-1-negative volunteers of trial HIV-CORE 002, who received either the CM (top) and DDDCM (bottom) vaccine regimen, are shown separately. Time points ‘S’ (screen) and 0–28 weeks were previously published [68] and are shown for completeness. ‘1y’ and ‘2y’ indicate 1 and 2 years after the last M (MVA.HIVconsv) vaccine administration at weeks 8 (CM) and 20 (DDDCM) indicated below the graphs. Volunteers’ ID numbers are shown on the graph legend. (C) Fresh *ex vivo* net total IFN-γ ELISPOT assay frequencies of HIVconsv-specific T cells after 1 and 2 years. The horizontal bars represent median frequencies for each regimen separately. The two time points and regimens were not statistically separable.

Healthy, low risk HIV-1-negative adults received T-cell immunogen HIVconsv [55] (Fig 1A) derived from conserved regions of the HIV-1 proteome in trial HIV-CORE 002, which took place in Oxford, UK between March 2011 and April 2015 [68, 71]. HIVconsv was delivered using plasmid DNA as pSG2.HIVconsv (D), engineered non-replicating simian adenovirus as ChAdV63.HIVconsv (C) and non-replicating poxvirus modified vaccinia virus Ankara as MVA.HIVconsv (M) combined into heterologous regimens. Total frequencies of vaccine-induced and persisting HIVconsv-specific CD4^+^ and CD8^+^ T cells were determined in an IFN-γ ELISPOT assay using 6 pools of 15-mer peptides overlapping by 11 amino acids, which together spanned the entire HIVconsv protein (Fig 1A).

The HIV-CORE 002 trial protocol monitored volunteers’ responses until week 28. To determine longevity of the vaccine-elicited T cells and assess any possible benefits, or their absence, of the plasmid DNA vaccine priming, volunteers, who received the two most potent regimens CM and DDDCM, were invited to provide blood samples at 6 months, and at 1 and 2 years after the last vaccine administration. Of the 16 subjects in total, 12 and 8 provided blood samples at 1 and 2 years post vaccination, respectively. At both visits, all vaccine recipients had detectable HIVconsv-specific responses in an *ex vivo* IFN-γ ELISPOT assay (Fig 1B). As the two CM and DDDCM regimens were not statistically separable at least in terms of the frequencies of HIV-1-specific, IFN-γ-producing T cells (Fig 1C) detected in an *ex vivo* ELISPOT assay, their frequencies were combined and gave median (range) of 990 (150–2495) and 763 (70–1745) SFU/10^6^ PBMC at 1 and 2 years, respectively. At year 1, the detected responses were broad with median 5 recognized peptide pools (frequencies above 50 SFU/10^6^ PBMC above the no-peptide background) out of 6 pools in total.

### Long-lived HIVconsv-specific cells show broad functional profile

**Fig 2:**
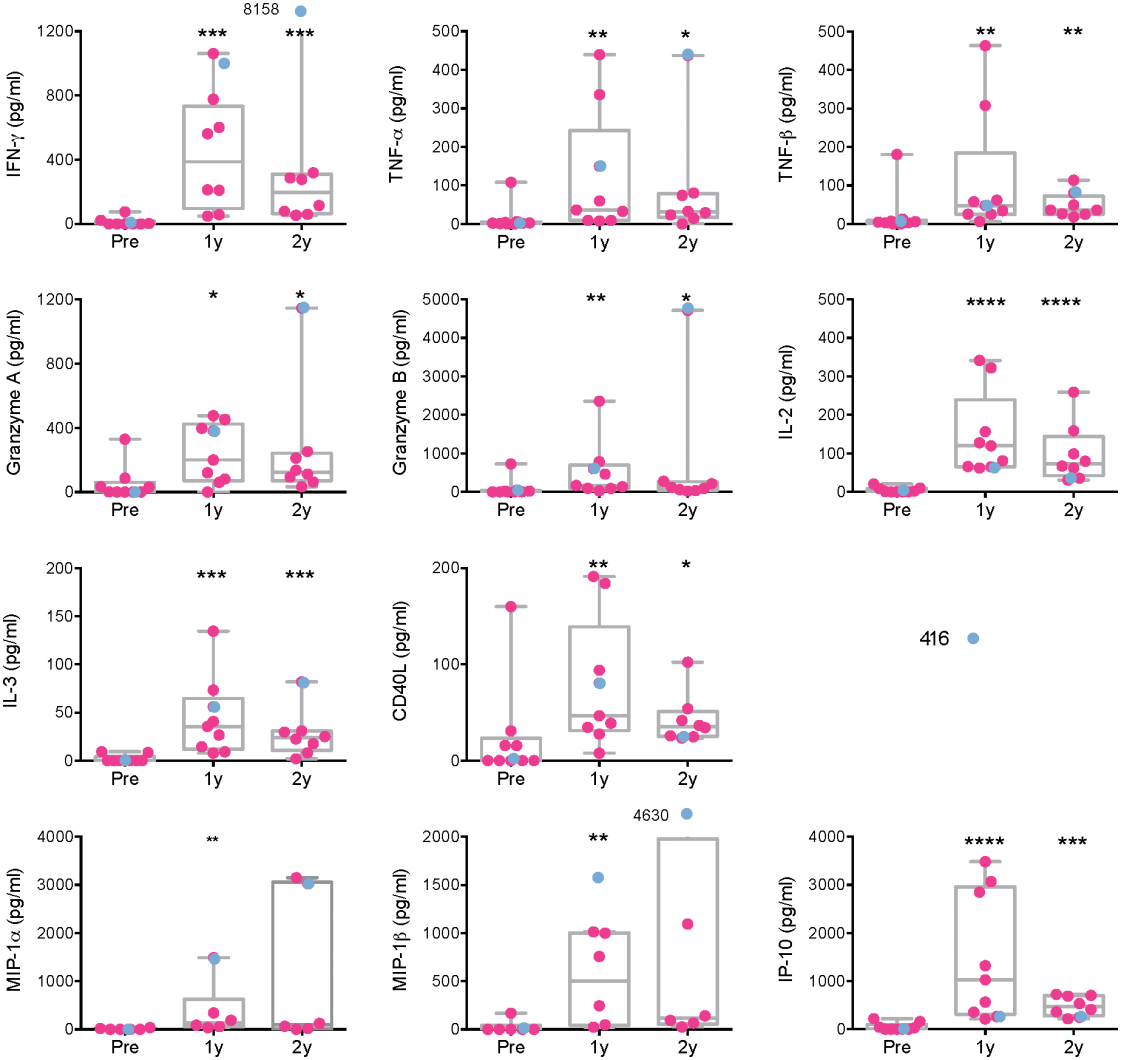
Long-term broad functional capacity of HIVconsv vaccine-elicited human T cells. Cryopreserved and thawed PBMC samples from pre-vaccination (Pre), and 1 (1y) and 2 (2y) years after the last vaccine administration were re-stimulated with pools of stimulatory 15-mer peptides assembled for each returning volunteer individually for 48 hours and the tissue culture supernatants were analyzed in a 25-analyte Luminex assay. Individual values are shown with indicated median and boxed interquartile range. Volunteer 416 is depicted in blue. Only cytokines and chemokines with positive responses are shown. Results were analyzed using the Mann-Whitney U test for comparison between the long-term samples vs the pre-vaccination sample. Significant *P* values are indicated by asterisks, whereby: *—less that 0.05; **—less than 0.01; ***—less than 0.001; ****—less than 0.0001.

**Fig 3:**
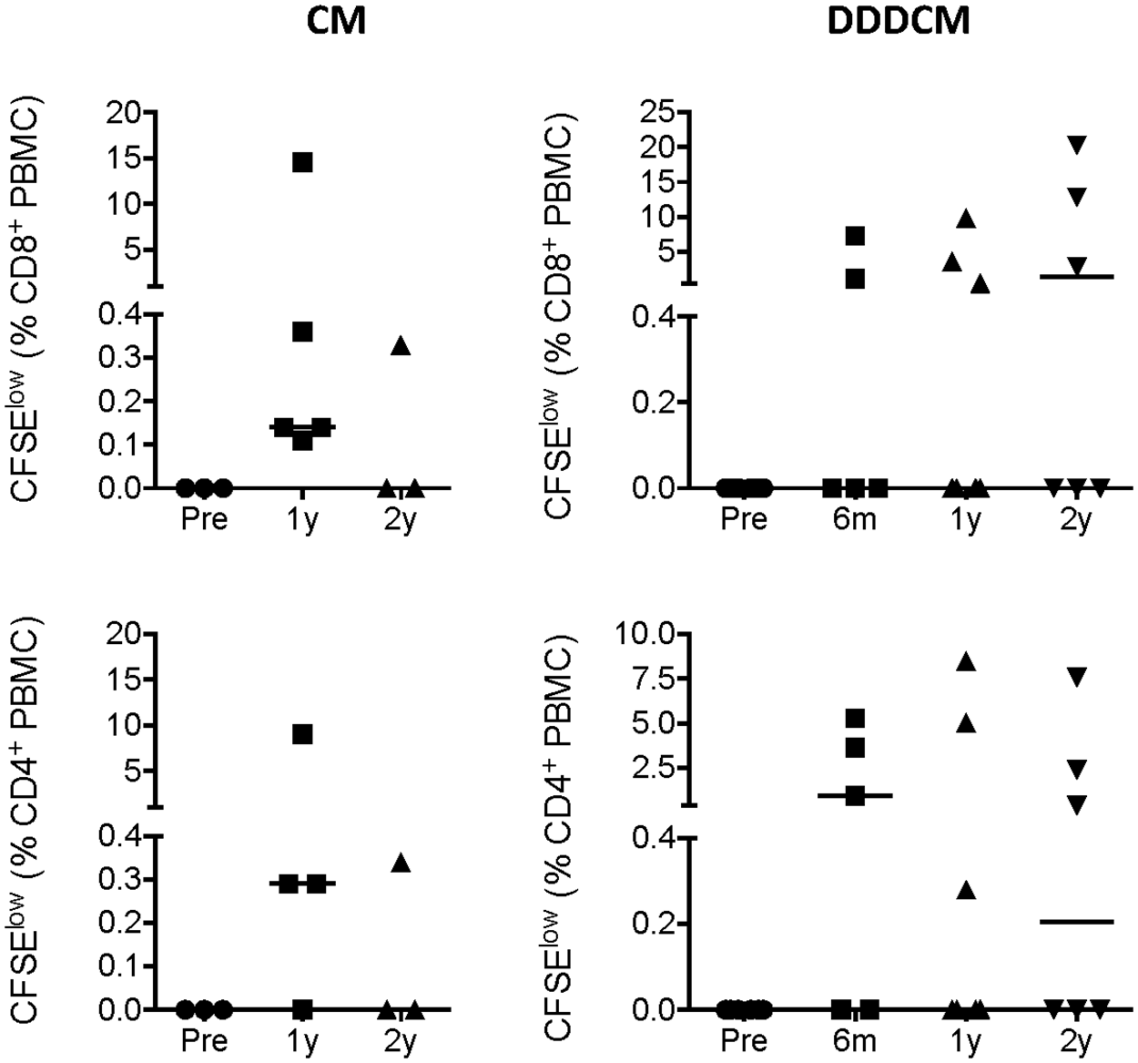
Proliferative capacity of long-term vaccine-elicited human T cells. Cryopreserved and thawed PBMCs from pre-vaccination (Pre), 6 months (6m), and 1 (1y) and 2 (2y) years after the last vaccine administration were labelled with CFSE, stimulated with pools of stimulatory 15-mer peptides assembled for each returning volunteer for 5 days and analyzed using flow cytometry for dividing (decreased CFSE) CD4^+^ and CD8^+^ T cells. The CM and DDDCM vaccination regimens are indicated above. The gating strategy is given in S1 Fig. Individual values are shown with median as a horizontal line.

Next, we assessed the functionality of the long-lived vaccine-induced T cells. For each volunteer, a personalized set of 15-mer peptides was assembled based on the mapping of stimulatory peptides performed previously [61–63]. Volunteers’ cryopreserved and thawed PBMCs were incubated with a personalized peptide pool for 48 hours and the tissue culture supernatants were analyzed for 25 intercellular signaling factors using a Luminex assay. Overall, low varied levels of a range of cytokines and chemokines were detected, which included those classified as T_H_1 (IFN-γ, TNF-α, TNF-β, IL-2 and granzymes A and B), T_H_2 (IL-1 and CD40L), with *in vitro* noncytotoxic HIV-1 inhibition (MIP-1α/CCL3, MIP-1β/CCL4) and IP-10/CXCL10 (Fig 2). Using CFSE-dilution assay (S1 Fig), approximately half of the PBMC samples proliferated upon specific peptide restimulation within the 5 days of the assay (Fig 3), which could reflect possible differences in the kinetics of the responses. The main reason for not detecting proliferation in some samples was the very low frequency of peptide-specific cells. Thus, low, but definite frequencies of persisting vaccine-elicited plurifunctional T-cell populations were maintained in the circulation of the HIVconsv-vaccine recipients for at least 2 years.

### Both CD8^+^ and CD4^+^ memory T cells persisted

**Fig 4:**
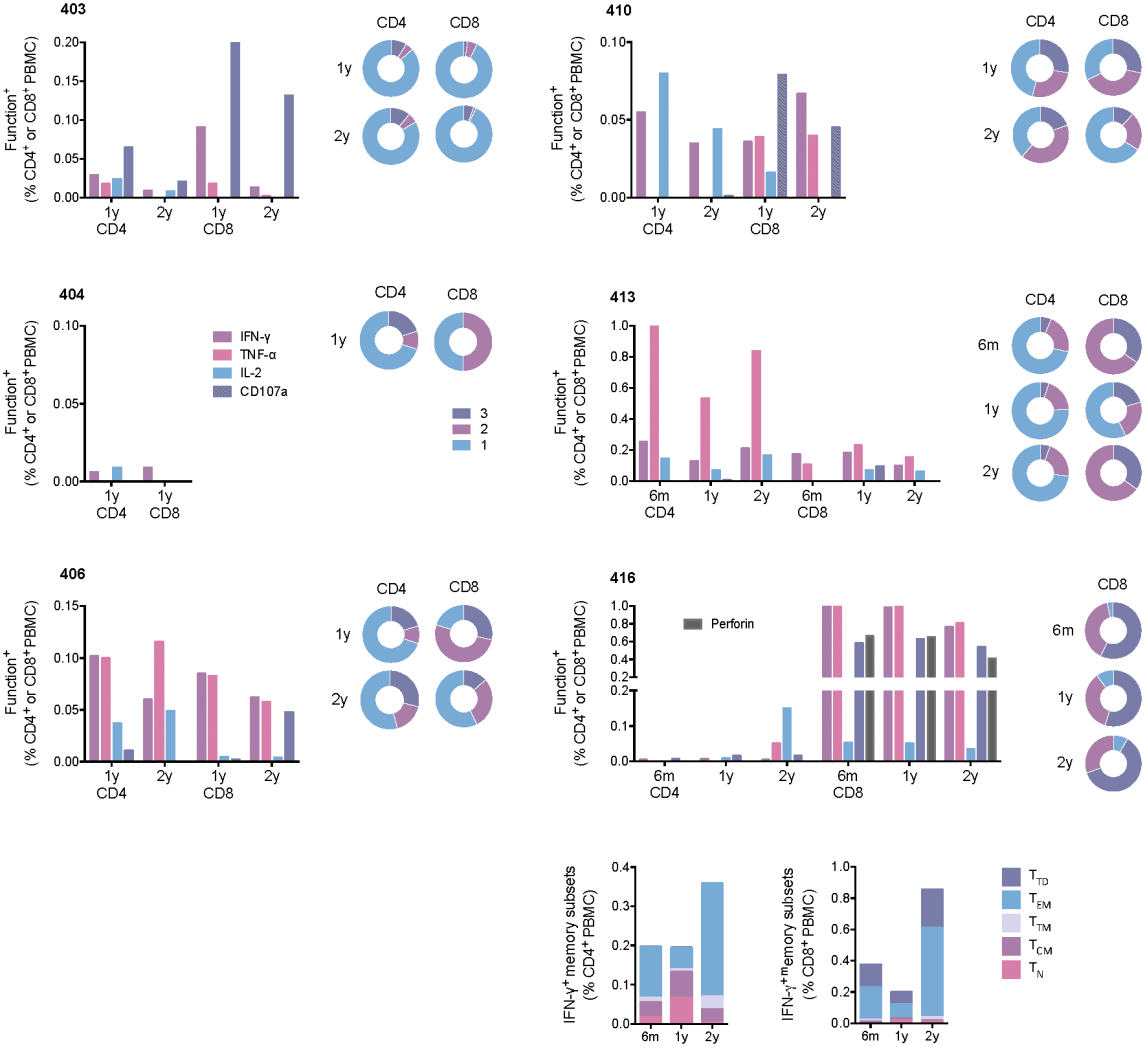
Functionality and memory subtypes of vaccine-elicited human CD4^+^ and CD8^+^ T cells. Frozen and thawed PBMCs from 6 months (6m), and 1 (1y) and 2 (2y) years after the last vaccine administration were stimulated with personalized 15-mer peptide pools and subjected to ICS assay. The pie charts refer to the plurifunctionality of the responses defined by Boolean gating. The results from six individuals are shown. For the best responder 416, memory phenotypes were investigated by flow cytometry using IFN-⅟ release to identify antigen-specific cells. Memory subsets are defined as T_N_−naïve T cells (CD45RA^hi^CCR7^hi^CD27^hi^). T_CM_—central memory (CD45RA^lo^CCR7^hi^CD27^hi^), T_TM_—transitional memory (CD45RA^lo^CCR7^lo^CD27^hi^), T_EM_—effector memory (CD45RA^lo^CCR7^lo^CD27^lo^), and T_TD_—terminally differentiated (CD45RA^hi^CCR7^lo^CD27^lo^).

For six vaccine recipients with sufficiently high frequencies of HIVconsv-specific T cells, intracellular cytokine staining assay (ICS) was performed to characterize separately persisting CD4^+^ and CD8^+^ T cells (see S1 Fig for the gating strategy). Overall in the tested subjects, absolute and relative frequencies of CD4^+^ and CD8^+^ T cells varied, decreased with time to below 1% of total peripheral CD4^+^ or CD8^+^ T cells and displayed plurifunctionality in terms of IFN-γ, TNF-α and IL-2 expression and degranulation revealed by CD107a surface expression upon personalized peptide pool re-stimulation (Fig 4). At one end of the spectrum, subject 413 maintained high frequencies of 0.84% of TNF-α-producing CD4^+^ cells by 2 years post-vaccination, while his/her HIVconsv-specific CD8^+^ cells decreased to approximately 0.2% of total CD8^+^ PBMCS. In contrast at same time point, volunteer 416 had high level of HIVconsv-specific CD8^+^ T-cells still at 0.8% of total CD8^+^ PBMCS, while his/her CD4^+^ cells were down to 0.2% of total CD4^+^ PBMCS. In subject 416, we also noted a good agreement of the CD107a marker with the perforin cell content. For this subject, we also used a panel of anti-CD45RA, anti-CCR7 and anti-CD27 mAbs to profile the T-cell memory subsets. At the 2-year time point, the predominant CD4^+^ and CD8^+^ subsets were effector memory T cells (CD45RA^lo^CCR7^lo^CD27^lo^). While CD8^+^ cells had as the second most abundant subpopulation terminally differentiated memory T cells, which reverted to CD45RA positivity (CD45RA^hi^CCR7^lo^CD27^lo^), CD4^+^ showed smaller, equally abundant fractions of transitional (CD45RA^lo^CCR7^lo^CD27^hi^) and central (CD45RA^lo^CCR7^hi^CD27^hi^) memory T cells (Fig 4 bottom). All populations were negative for the programmed cell death protein 1 (PD-1)/CD279 and T-cell immunereceptor with Ig and ITIM domains (TIGIT) inhibitory markers (S2 Fig). Thus, the polychromatic flow cell analysis revealed overall plurifunctional HIVconsv-specific human memory T cells maintained for 2 years post-vaccination.

### HLA-peptide dextramer analysis confirmed plurifunctionality and predominant effector memory T-cell subset

**Fig 5:**
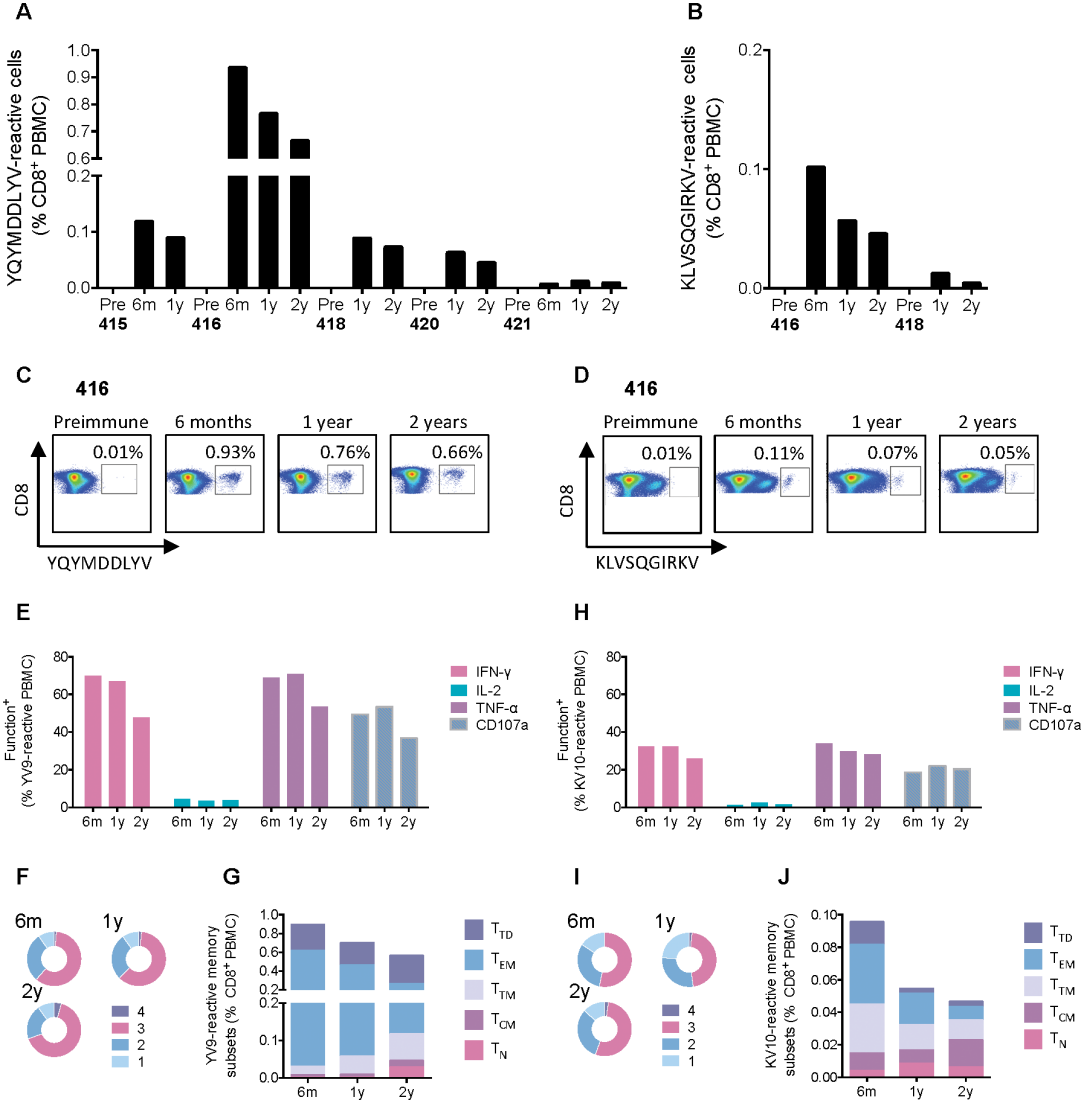
Dextramer-aided analysis of human YV9- and KV10-specific CD8^+^ T cells. For HLA-A*02:01-positive vaccine recipients, frozen and thawed PBMCs from 6 months (6m), and 1 (1y) and 2 (2y) years after vaccination were incubated with HLA-A*02:01-YV9 (A and C) or HLA-A*02:01-KV10 (B and D) dextramers together with other cell-surface markers and analyzed using flow cytometry (S3 Fig). Alternatively, PBMCs of subject 416 were stimulated with personalized 15-mer peptide pools and reacted with a panel of functional mAbs together with HLA-A*02:01-YV9 (E and F) or HLA-A*02:01-KV10 (H and I) dextramers. The pie charts (F and I) show the plurifunctionality of dextramer positive CD8^+^ T cells. PBMCs from subject 416 reactive with the HLA-A*02:01-YV9 (G) or HLA-A*02:01-KV10 (J) dextramers were phenotyped for memory subsets defined as T_N_−naïve T cells (CD45RA^hi^CCR7^hi^CD27^hi^). T_CM_—central memory (CD45RA^lo^CCR7^hi^CD27^hi^), T_TM_—transitional memory (CD45RA^lo^CCR7^lo^CD27^hi^), T_EM_—effector memory (CD45RA^lo^CCR7^lo^CD27^lo^), and T_TD_—terminally differentiated (CD45RA^hi^CCR7^lo^CD27^lo^) (S3 Fig).

Five of the returning vaccinees carried the HLA-A*02:01 allele. Taking advantage of our fine epitope mapping in this trial population [63], we used a dextramer of HLA-A*02:01-peptide complexes to analyze YQYMDDLYV (YV9) and KLVSQGIRKV (KV10) epitope-specific CD8^+^ T cells. Both epitopes originate from the HIV-1 Pol polyprotein. Thus, using the most immunodominant epitope YV9 of the two, which spans the active site of the reverse transcriptase and, therefore, is highly conserved across diverse HIV-1 variants and capable of mediating inhibition of HIV-1-replication *in vitro* [61], the five volunteers ranked in the order of (from the highest) 416, 415, 418, 420 and 421 with ranging frequencies of 0.66% to 0.08% of dextramer-reactive cells per CD8^+^ T cells at the 2-year re-bleed (Fig 5A). T cells specific for the weakest epitope KV10 were tested in volunteers 416 and 418, and were of respective 0.05% and 0.01% of CD8^+^ T cells at year 2 (Fig 5B). For subject 416, the frequencies of both the YV9- and KV10-specific cells decreased steadily from 6 months to 2 years after vaccination, maintaining for the strongest YV9 responses at 0.93% to 0.76% to 0.66% of total CD8^+^ cells, respectively (Fig 5C and 5D). Relatively high frequencies of dextramer-reactive cells in subject 416 allowed definition of their functional and memory subsets. Thus, approximately two-thirds of the YV9-specific cells were tri-functional for the production IFN-γ, TNF-α and CD107a (Fig 5E and 5F). Proportions of the effector memory T-cell subset decreased with time and ended balanced with fractions of terminally differentiated, transitional and naive memory subsets at 2 years (Fig 5G). The results are similar for CD8^+^ T cells recognizing the subdominant epitope KV10 with about one-half of tri-functional cells (Fig 5H and 5I), diminishing effector memory subset with time and more even representation of memory subsets at year 2 (Fig 5J). Both YV9- and KV10-specific cells were PD1- and TIGIT-negative (S2 and S3 Figs). Thus, analysis of the dextramer-reactive CD8^+^ T-cells found desirable properties for a vaccine-elicited long-lived memory T cells and concurred with the above observations on the HIVconsv-specific CD8^+^ T-cell population. Given the expected more gradual continuous changes over the three time points of the specific phenotypic subsets, the dextramer analysis likely provides more sensitive, accurate and reproducible measurements compared to the global CD8^+^ T-cell evaluation.

## Discussion

In the present work, we studied the longevity of T-cell responses against HIV-1 induced by conserved-region vaccines in trial HIV-CORE 002, which recruited healthy adults of low risk of HIV-1 infection in the UK [68]. The first generation immunogen HIVconsv [55] utilizes 14 conserved regions of alternating-clade-consensus amino acid sequences, which were delivered by plasmid DNA (D), simian adenovirus ChAdV-63 (C) and MVA (M) in heterologous regimens. As for the response magnitude, all 12/12 returning subjects at year 1 and 8/8 subjects at year 2 visits after vaccination had detectable HIVconsv-specific T-cell responses. Frequencies of specific T-cells did not differ statistically between the CM and DDDCM investigated regimens and thus were merged for further data analyses. While the combined CM- and DDDCM-elicited HIVconsv-specific T-cell frequencies peaked at median 5,170 IFN-γ SFU/10^6^ PBMC [68], they declined to median of 990 SFU/10^6^ PBMC over 1 year and 763 SFU/10^6^ PBMC over 2 years post vaccination, which corresponds to respective 19% and 15% of the peak frequency. In comparison, frequencies reported in the STEP study at peak ranged 163–686 IFN-γ SFU/10^6^ PBMC [72].

The vaccine-elicited T-cell responses persisting in subjects’ PBMCs produced an array of cytokines and chemokines released into the culture supernatants upon HIVconsv peptide stimulation, which included Th1- and Th2-associated factors as well as chemokines *in vitro* directly involved in HIV-1 replication inhibition. Overall, the ex vivo ELISPOT, Luminex and ICS assays correlated very well with each other (S4 Fig). The levels of persisting CD4^+^ and CD8^+^ T cells measured in the ICS assay ranged from 0.01% to 1% of the total T-cell subset frequencies, which is broadly comparable with the ICS frequencies reported (for earlier time points) in the other T-cell vaccine studies [67, 72, 73]. The frequencies and plurifunctionality of HIVconsv-specific CD8^+^ T cells using HLA-A*02:01-peptide dextramers concurred well with those observed in the polychromatic ICS analysis and, for functionality, with the Luminex assay. Direct detection and characterization of responses by dextramers offers rapid, sensitive and reproducible measurements of plurifunctionality on a single cell level. While in the past, initial simple inverse correlation between frequency of HLA-peptide tetramer-reactive cells and plasma viral load was reported [15], this was not detected in several later studies [74–76] most likely due to the specificity and heterogeneity of the studied T cells discussed above. Similarly, while the first demonstration of plurifunctionality of human CD8^+^ T cells presented a possibility that certain functional pattern(s) may be associated with control of HIV-1 [3, 77], these are not likely to be narrowed down without paying attention to T-cell specificity and the other critical parameters. Thus, the requirement of plurifunctionality of CD8^+^ T cells for HIV-1 protection makes sense, but warrants further investigations.

As expected in healthy volunteers, the T cell-memory-subset analysis of vaccine elicited responses at 1 and 2 years after vaccination revealed structured subpopulations free of PD-1 and TIGIT inhibition markers. While central memory cells (CCR7^hi^) recirculate through the blood and secondary lymphoid organs, effector memory cells (CCR7^lo^) transit through blood and peripheral tissues [78–80]; both populations can be further subdivided by expression of CD27, whereby only the CD27^hi^ cells exhibit optimal recall proliferation and self-renewal potential [81, 82]. However, the links between the phenotypic CD8^+^ T cell-memory structure to protective efficacy against pathogens is only emerging [81–83]. HIVconsv vaccines induced both CD8^+^ and CD4^+^ memory T cells. This is important as CD4^+^ T cells provide pivotal helper signals for the generation of memory and longevity of CD8^+^ T-cell responses. Furthermore, about one-third of IFN-γ^+^ CD4^+^ T cells were reported to also have a cytotoxic functional phenotype [84] and directly kill HIV-1-infected cells *ex vivo* [85].

Overall, the HIVconsv immunogen delivered by the CM and DDDCM regimens induced memory CD4^+^ and CD8^+^ T cells of desirable features lasting at least 2 years after vaccine administration. The significance of these observations in terms of *in vivo* suppression of HIV-1 replication can only be established by assessing whether or not and to which extent vaccinees exposed to HIV-1 are protected from persistent infection and/or immunodeficiency.

## Supporting information

S1 Fig Gating strategy for T-cell proliferation assay by CFSE dilution. (PDF)

S2 Fig Gating strategy for detection of HIVconsv-specific memory T-cell subsets using IFN-γ capture assay in PBMCS of HLA-A*02:01-positive vaccine recipients. (PDF)

S3 Fig Gating strategy for detection of memory CD8^+^ T-cell subsets within dextramer-reactive PBMCS. (PDF)

S4 Fig Correlations between Luminex, ICS, ELISPOT and CFSE proliferation assays. (PDF)
