# Supplementary figures and images for: Long-term follow up of human T-cell responses to conserved HIV-1 regions elicited by DNA/simian adenovirus/MVA vaccine regimens
Source: PLoS One. 2017 Jul 18;12(7):e0181382. doi: 10.1371/journal.pone.0181382 (PMC5515449; doi:10.1371/journal.pone.0181382)

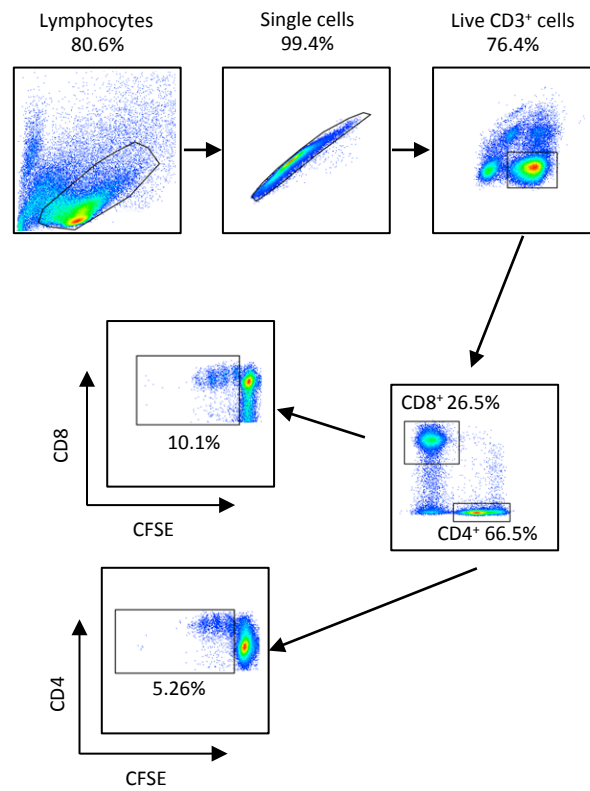

S1 Fig. Gating strategy for T-cell proliferation assay by CFSE dilution.

Supplement: S1 Fig — (PDF) [file pone.0181382.s001.pdf]
