# Supplementary material for: Long-term follow up of human T-cell responses to conserved HIV-1 regions elicited by DNA/simian adenovirus/MVA vaccine regimens
Source: PLoS One. 2017 Jul 18;12(7):e0181382. doi: 10.1371/journal.pone.0181382 (PMC5515449; doi:10.1371/journal.pone.0181382)
Supplement: S2 Fig — (PDF) [file pone.0181382.s002.pdf]

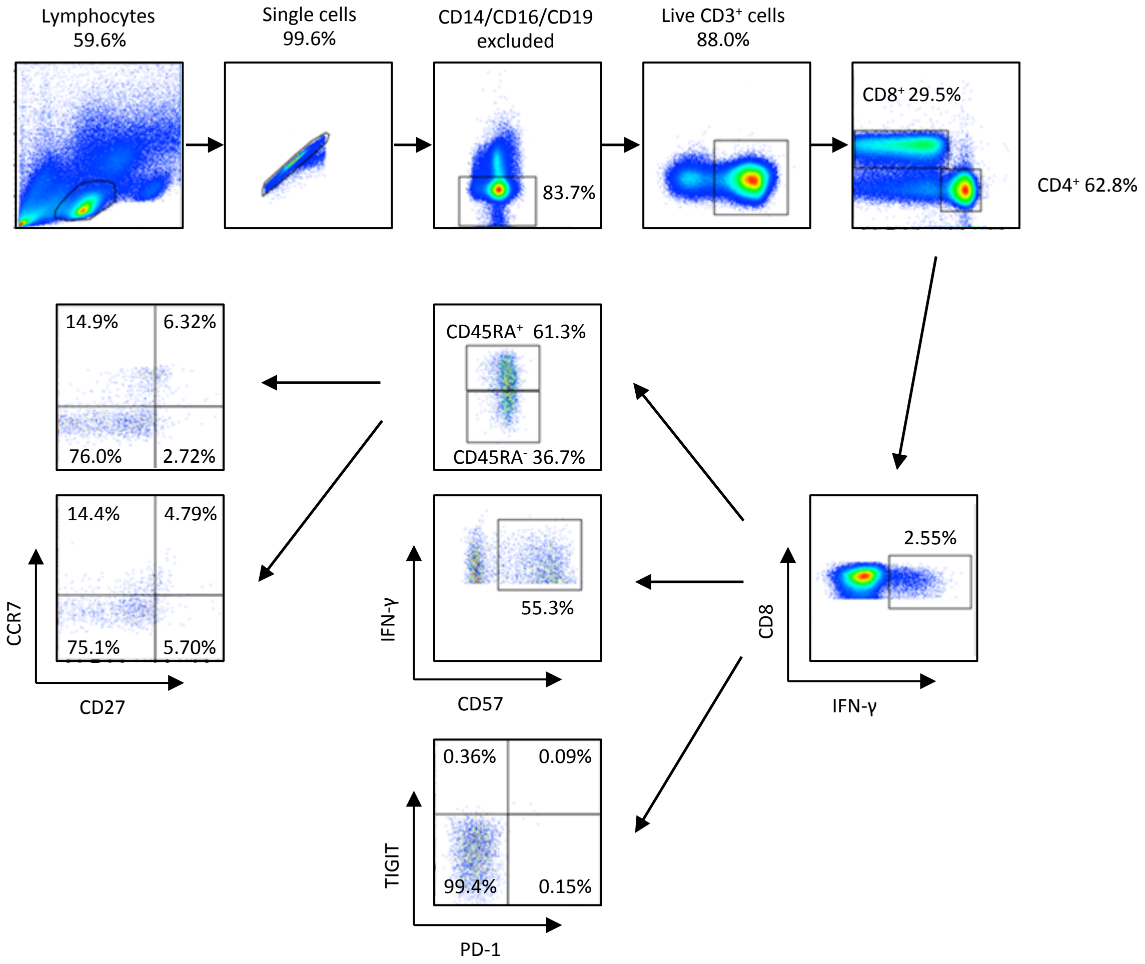

S2 Fig. Gating strategy for detection of HIVconsv-specific memory T-cell subsets using IFN-γ capture assay in PBMCs of HLA-A*02:01-positive vaccine recipients.
