# Supplementary material for: Long-term follow up of human T-cell responses to conserved HIV-1 regions elicited by DNA/simian adenovirus/MVA vaccine regimens
Source: PLoS One. 2017 Jul 18;12(7):e0181382. doi: 10.1371/journal.pone.0181382 (PMC5515449; doi:10.1371/journal.pone.0181382)
Supplement: S3 Fig — (PDF) [file pone.0181382.s003.pdf]

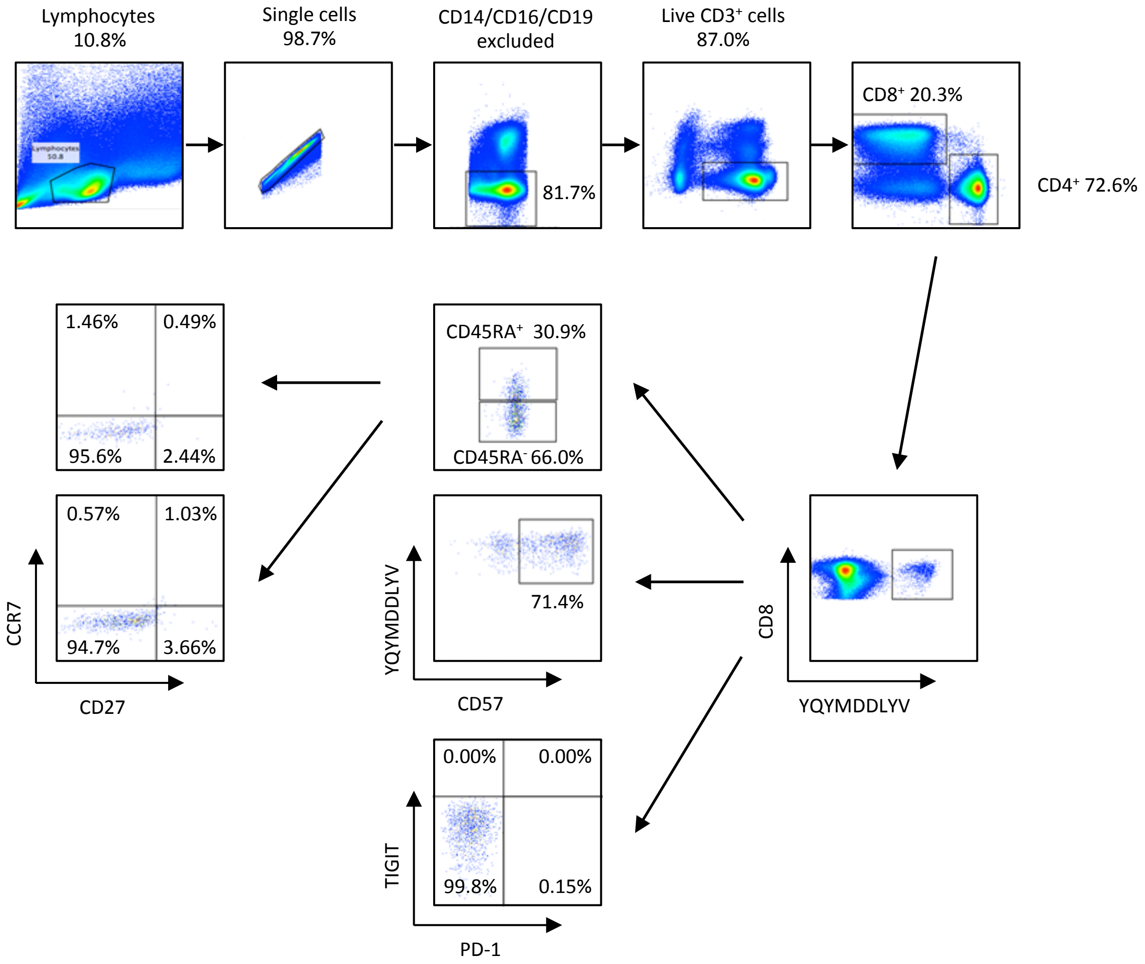

S3 Fig. Gating strategy for detection of memory CD8+ T-cell subsets within dextramer-reactive PBMCs.
