# Supplementary material for: Long-term follow up of human T-cell responses to conserved HIV-1 regions elicited by DNA/simian adenovirus/MVA vaccine regimens
Source: PLoS One. 2017 Jul 18;12(7):e0181382. doi: 10.1371/journal.pone.0181382 (PMC5515449; doi:10.1371/journal.pone.0181382)
Supplement: S4 Fig — (PDF) [file pone.0181382.s004.pdf]

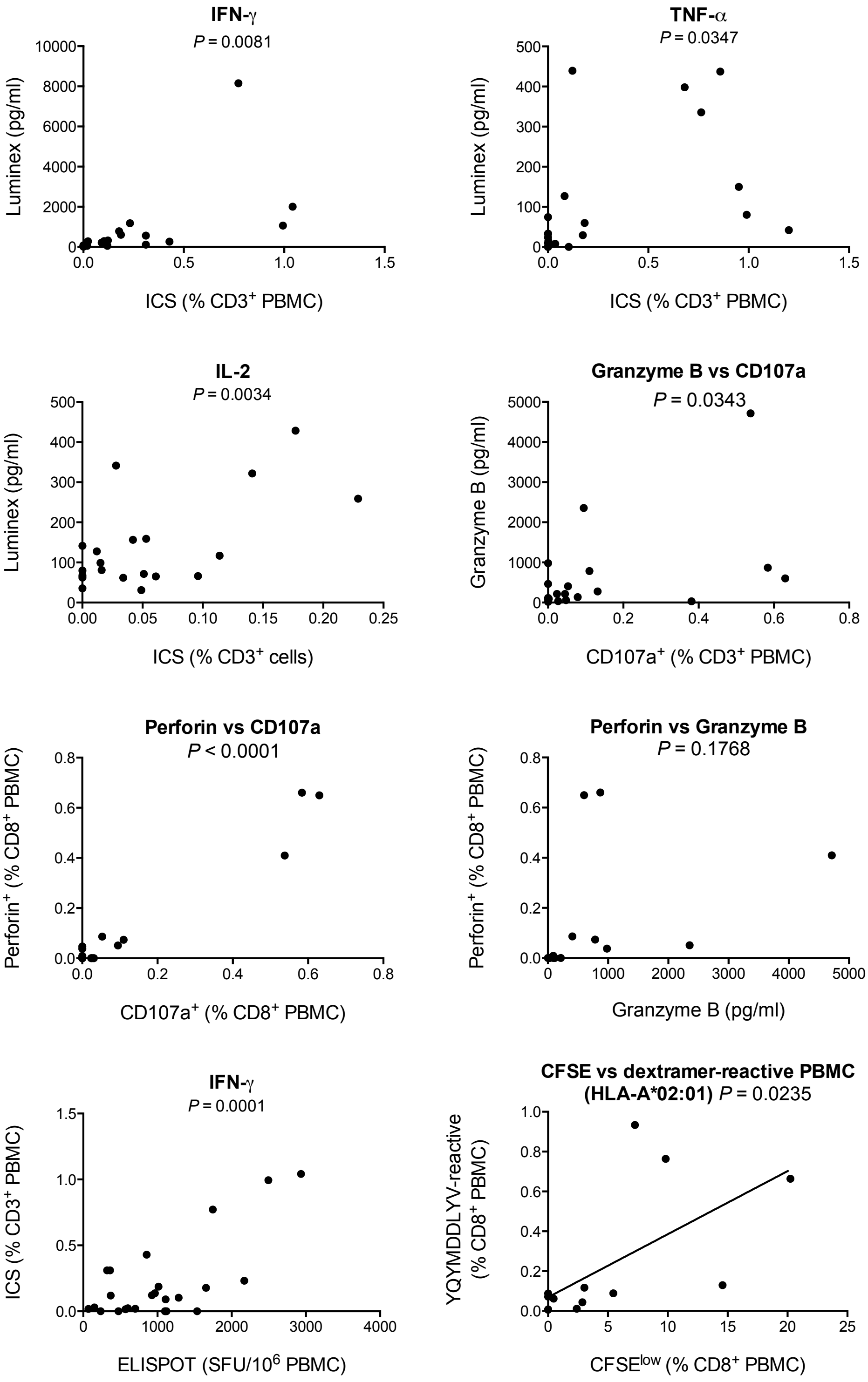

S4 Fig. Correlations between Luminex, ICS, ELISPOT and CFSE proliferation assays. Volunteers' PBMCs were restimulated using personalized 15-mer peptide pools in either Luminex, ICS, ELISPOT or CFSE prolifeation assays as described in Materials and Methods. Correlation significance was determined using the Prism 6 software and *P* values (two-tailed) are given in the title of each graph.
